# A Transgender Studies Approach for Educators in Schools: Reflections on “Cissexist Pitfalls,” Bifurcated Frameworks, and Racial Justice

**DOI:** 10.1177/01614681221121514

**Published:** 2022-08-26

**Authors:** Wayne Martino

**Affiliations:** 1 The University of Western Ontario, London, ON, Canada

**Keywords:** cissexism, educators in schools, fugitive justice, teacher professional learning, trans epistemic justice, trans studies, white supremacy

## Abstract

**Background/Context:**

Trans studies provides onto-epistemological, theoretical, ethical, and political frameworks that have a particular application for studies in education, and specifically for educators in schools, that remains largely unexplored or unelaborated. Within a context of resurgent right-wing extremism that fuels anti-trans and white supremacist rhetoric, trans studies provides analytic tools for deepening an understanding of gender expansive education and addressing gender and racial justice in schools.

**Purpose/Focus of Study:**

The purpose of this article is to illuminate the utility of trans-informed frameworks and the hermeneutic resources they provide in their potential to enhance and deepen an understanding of the pedagogical interventions in the classroom that are needed to educate about trans marginalization and racial justice. The focus is on the application of these frameworks, both with respect to fostering professional learning for educators in schools, and for my own pedagogy and course development.

**Research Design:**

In adopting a critical incident/critical reflexive practitioner approach, the author provides a sustained reflection on his own pedagogical practices and approach to curriculum development within the context of teaching a graduate course on gender and sexual diversity in schools. While such critical reflections emerged in response to one core critical incident, they are extended through an application of a trans studies approach to thinking through the scaffolded learning process for educators in the application of trans studies, and what this means for the sort of teacher threshold knowledges that are needed in schools for addressing gender and racial justice.

**Findings:**

Trans studies provides hermeneutic resources and analytic concepts that serve as tools for supporting teachers in reflecting on cissexist assumptions and how these materialize through their practice in the classroom. A concretization of what it means for teachers to enact a trans-studies-informed pedagogy requires a scaffolded approach and one that entails a weaving through of a critical and integrated focus on race, settler colonialism, and cissexism.

**Conclusions:**

The article highlights that there are certain limits to conceiving of a pedagogical commitment to interrogating gender binaries as a basis for addressing gender-expansive education in schools. The author concludes that such frameworks, in an absence of embracing a trans-informed approach to thinking about gender diversity and racial justice, run the risk of relying on a bifurcating categorization of trans students’ identities that ignore fundamental questions related to the impact of cissexism and its implication in colonial domination and racialization.

## Introduction

In this article, I reflect on the necessity of embracing a trans studies–informed and critical trans political approach to enacting gender and racial justice in schools. Along with my colleagues, I have been arguing for this sort of engagement with trans studies on epistemological and ontological grounds, raising theoretical, ethical, and empirical questions about the sort of frameworks and knowledge that are needed to enhance a deeper understanding of gender identity and gender expression in schools and educational research (Blackburn et al., 2016; Cumming-Potvin & Martino, 2018a, 2018b; Kuhl & Martino, 2018; Martino, 2016; Martino & Cumming-Potvin, 2015, 2018, 2019; Martino & Ingrey, 2020; Martino & Omercajic, 2021; Martino, Kassen, & Omercajic, 2022; Omercajic & Martino, 2020).^1^ In this article, I extend this work in focusing on my own experiences as a cis white gay male instructor in teaching a graduate course on sexual diversity and trans-affirmative education. The course is part of a professional program for educators that is devoted to addressing equity and social justice in schools. My experience has helped me to think through the ethical and political implications of envisaging the curriculum as a site for redressing epistemic injustice that goes beyond merely a commitment to recognition and representation “as a primary pedagogical model for the social treatment of transgender people” (Keegan, 2022, p. 26; also see Malatino, 2015; Nicolazzo, 2017). Fricker (2007), for example, refers to testimonial injustice as a form of epistemic injustice that occurs when there is a failure to allow for the individual’s self-determining capacity to generate and share knowledge about their own identity, lived experiences, and understandings of their personhood. The result, she argues, is to *deflate* or, rather, deny one’s credibility as a knower, which produces both erasure and delegitimation of one’s self-knowledge. However, Fricker notes that testimonial injustice is inextricably tied to hermeneutic injustice in that the provision of knowledge and frameworks for validating and making sense of one’s identity is not readily or officially available. This injustice is manifested in the systemic failure to provide trans-affirming and gender expansive education in schools and preservice teacher education, which results in “some significant area of one’s social experience obscured from collective understanding owing to a structural identity prejudice in the collective hermeneutic resource” (Fricker, 2007, p. 155; also see Steele & Nicholson, 2020). Kennedy (2018), for example, refers to such systemic forces that contribute to thwarting both testimonial and hermeneutic justice for trans people as *cultural cisgenderism*, which “represents a systemic erasure and problematizing of trans people and the experiences between trans and cisgender people [that lead to] essentializ[ing] sex/gender as biologically determined, fixed at birth, immutable, natural and externally imposed on the individual” (Riggs & Bartholomaeus, 2018, p. 308). However, central to addressing such epistemic injustice in the classroom, I argue, is the need to build teacher threshold knowledges for educators about the ways in which other forms of resistance and struggles inspired by decolonial and Two-Spirit critiques of settler colonialism, as well as a trans critique of racialized gender systems of inequality, can be a powerful source of pedagogical inspiration for building a more robust understanding of trans marginalization.

My purpose in writing this article, therefore, is to first illuminate the need for trans-informed interpretive frameworks that center a critique of cisgenderism as central to enacting an ethical and political commitment to addressing trans marginalization in schools. My aim is to explicate what these frameworks might mean pedagogically for educators in schools in terms that support testimonial and hermeneutic justice for trans students and for envisaging gender-expansive education in schools (Martino & Omercajic, 2021; skelton, 2022). I then go on to elaborate on a more sustained engagement with the work of trans scholars in terms of reflecting on questions related to building teacher knowledge that supports the cultivation of teacher professional judgement and learning as it pertains to enacting a gender-expansive curriculum committed to racial justice. I draw attention to the necessity of *refusing* the imposition of binary thinking about trans people as a normalizing tendency that entails imposing a categorical differentiation between binary and nonbinary trans people that does not account for or do justice to trans people’s “own understanding of their genders” (Ansara & Berger, 2016, p. 2). The focus is on thinking through important ethical questions related to a commitment to centering the experiences and voices of trans people—what Stryker (2006) refers to as trans desubjugation—in the pedagogical space of the classroom and what this means for envisaging a critical trans politics in schools. Stryker has identified the importance of addressing “questions of embodied difference” and “transgender phenomena” as they pertain to “the operations of systems and institutions that simultaneously produce various possibilities of viable personhood, and eliminate others” (p. 3). Butler (2004), for example, argues that the

critique of gender norms must be situated within the context of lives as they are lived, and must be guided by the question of what maximizes the possibilities for a livable life, what minimizes the possibility of an unbearable life or indeed, social or literal death. (p. 8)

Hence, this commitment to trans desubjugation and self-determination needs to involve being cognizant of what Radi (2019) referred to as the “epistemic disavowal” of trans and gender diverse people’s understanding of their own personhood and embodied experiences. It also needs to embrace an ethic of “respectful curiosity” (Raun, 2014) in fostering pedagogical understandings of transness in the classroom that does justice to supporting the self-determination of trans people—especially given the history of epistemological violence (Teo, 2010) that has been enacted against them (Kuhl, 2019; Kuhl & Martino, 2018; Tosh, 2016), and the historical legacy of “ongoing colonialism” and white supremacy (Driskill, 2010). Moreover, as Johnson (2015) highlights, such an ethical commitment also entails “avoiding cissexist analytical pitfalls” which inadvertently center and privilege “a reconstruction of the social from a cisgender perspective” (p. 26) that relies on a tendency to “project arbitrary meanings and value judgements onto different gendered and sexual bodies and behaviours” (Serano, 2013, p. 169, as cited in Johnson, 2015, p. 27).

## Cissexist Pitfalls in Thinking About Trans Students in the Classroom

I begin with a reflection on a *critical incident*^2^ that occurred within the context of a class discussion about supporting trans students in schools. The incident involved two teachers drawing a distinction between binary and nonbinary students in their classrooms. The incident occurred within the context of my teaching a graduate course on gender and sexual diversity; the course is part of an online program for supporting equity and social justice in schools, which also entails delivering online synchronous classes. The first part of the course is devoted to introducing students to queer theoretical frameworks and their pedagogical implications in the classroom, while the second part of the course introduces students to trans-affirming frameworks for understanding gender identity and trans marginalization in schools. The course spans 12 weeks, and the incident occurred in Week 11, after the students had been introduced to readings that addressed key concepts of cisgenderism (Ansara & Berger, 2016; Kennedy, 2018), cissexism (Serano, 2014), and gender creativity (Ehrensaft, 2016). Throughout the four weeks leading up to this particular incident, students were also provided with opportunities to view a range of videos about “interrogating gender norms” and creating gender-inclusive schools (Skurnick & Gender Spectrum, 2016; Tedx Talks, 2015), black trans life (TED, 2020), and other sources that featured a diversity of trans youth themselves talking about their lived experiences (Skylark Children Youth and Families, 2015; Wylde, 2015). The students had also been provided with opportunities to reflect on various resources for use in their classrooms and to learn about Western and settler colonial binary categorizations of gender and sexuality (Laing, 2021; YouthLine, 2020a), as well as 2SLGBTQ+ young people with intersectional identities (YouthLine, 2020b).^3^

The article by Frohard-Dourlent (2018) was one of the set readings for Week 11’s topic, “Trans Affirmative and inclusive Education.” The article reported on teachers’ responses to trans students in their demands for recognition, which, their research shows, often leads to positioning students to take charge of educating others about trans inclusion and gender diversity. The effect, as Frohard-Dourlent exposed, is to absolve educators of their responsibility to address institutional forces of cisnormativity and cisgenderism that contribute to trans marginalization in the first place, and that indeed render trans students invisible. It leads to trans students being delegated the task of undertaking gender expansive and trans-affirming education in an education system in which cisgender privilege and cissexism are deeply and institutionally embedded. These systemic forces contribute in significant ways to thwarting gender democratization and gender justice. They result in a failure to provide the necessary resources and support for teachers to integrate gender expansive education into the curriculum in ways that move beyond a politics of representation, recognition, and visibility to address important questions of redistributive justice (Fraser, 2009). Such questions pertain specifically to the need for a deeper understanding of trans marginalization, the livability of trans lives, and racial justice for all trans people (Gossett, 2017)—a point that I expand on later in explicating the terms of what moving beyond a Trans 101 approach needs to entail (Green, 2010). These considerations are particularly salient in a climate of resurgent anti-LGBTQI+ rhetoric that is fueled by white supremacist, transphobic, and homophobic right-wing extremism, particularly in the United States—though certainly not confined to this settler colonial nation (Boone, 2022; Campoamor, 2022; Collins & Madani, 2022).

The discussion started with one student—an elementary school teacher—arguing that adults can indeed create roadblocks for children in realizing themselves and that it is important for adults to be aware that, from a very young age, children are quite capable of knowing themselves and their identity. Her own experiences with a young trans girl who transitioned in the early years of schooling was evidence of this. In fact, in response to her reading of the Frohard-Dourlent article, she argued that it was up to educators to become aware of the *cisnormative barriers* that hamper such conditions for fostering both testimonial and hermeneutic justice in the classroom and schools for trans and “gender nonconforming” children. Moreover, she asserted that it is the removal of such barriers that will create more gender-facilitative spaces in schools (Luecke, 2018), ultimately contributing to supporting trans students “driving their own car” with respect to the self-realization and expression of their gender identities.

Another student, also a teacher, chimed in to support this student’s viewpoint, reiterating that there is an institutional responsibility to addressing cisnormative dress codes, ensuring bathroom access for trans and gender expansive students, and educating about pronoun use. She reiterated that schools need to educate along these lines and not just accommodate students so that more people can learn that gender identity “is not always fixed.” This teacher, given her own experiences as an educator in schools, emphasized system-level problems of ensuring that trans students’ self-assigned identities were adhered to through record-keeping and documentation that both honored their chosen name and avoided the microaggression of deadnaming, while at the same time maintaining a student’s right to privacy, especially if they were not out to their families. However, both students raised the question of trans and nonbinary legibility and visibility as an issue in their own experience, claiming that it is easier for educators to support *binary trans* students than those who identify as nonbinary or are gender nonconforming. The first student claimed that the reason for this was that already-existing knowledge about gender binary systems—what she referred to as *fixed* understandings about being a boy and being a girl—made it easier for teachers to support the trans student’s social transitioning to a boy or a girl. She felt that it was harder for teachers to support gender nonconforming students because there is not “a tidy set of social rules and norms to rely on” to inform their understanding of these students. The second student concurred, claiming that those students who identify as trans and “move from one identity to another” fit societal definitions of “what a boy or girl should be like and behave like,” making it easier for teachers who are confounded by what to expect when students identify as gender fluid.

This discussion opened a pedagogical space for interrogating the assumptions at play in the binary logic governing constructs about trans students and their identities that educators carry around with them and that expose the sort of cissexist pitfalls of this thinking. I posed this question for the class: What assumptions are being made when we draw comparisons between binary and nonbinary trans or gender fluid people in this way? What is at play here is what Johnson (2015), in drawing on Serano (2013), referred to as “cissexist double standards”; these double standards result in cis teachers in this particular case projecting certain value judgments about the fixity of trans identity for those students who identify as boys or girls by locating them in “a false binary,” leaving unquestioned the unmarked experience of cis students who identify as boys and girls. In this way, the incident enabled me to explicitly address these trans-informed theoretical constructs in their capacity to expose how such binary assumptions about, and categorizations of, trans people are not equally applied to thinking about cis students and cis people in general through posing this question: To what extent is the same set of assumptions applied to understanding the gender identity of cis girls and cis boys? Driving such pedagogical interventions about exposing the limits of cis thinking about trans people is what Johnson (2015) refers to as an instance of ciscentricity, which he further explains as a cissexist double standard that “allow[s] cisgender to remain an unmarked and unremarkable experience while transgender is highlighted as exceptional” (p. 34). In drawing on such trans hermeneutic resources, I was able to highlight that cis girls and cis boys are not necessarily thought about in terms of experiencing their gender identity as conforming to *a tidy set of gender norms*, and the same applies to trans girls and trans boys. So, is this how we are encouraged to think about cis boys and cis girls—as simply conforming to gender norms, which is then interpreted as an investment in a binary identity when applied to a trans boy/trans girl? Hence, a space was created in this pedagogical encounter for extending our reflection, as a class, on the need to interrogate the cissexist double standards that rely on reinforcing binary tropes about trans girls and trans boys that result in totalizing, overgeneralizing, and, ultimately, objectifying them (Johnson, 2015).

This discussion also provided the opportunity to reflect further on the application of key theoretical tools previously introduced to students in the course; these centered specifically on cis privilege and cissexism “as crucial concepts in making sense of experiences of marginalization” (Radi, 2019, p. 54; also Serano, 2014) and their impact on trans students in schools. Drawing on these frameworks also enabled me to draw attention to an approach to understanding trans identities that is “geared around a normative/subversive axis” (Radi, 2019, p. 51), which entails making distinctions between binary and nonbinary students in the classroom that result in a reification of the gender binaries. As Elliot and Roen (1998) have noted, such understandings also have their genesis in a “conformity/deviance” (p. 235) model that leads to distinguishing categories of trans individuals who are constructed as either supporting or contesting the gender order; the former are understood as “gender defenders,” maintaining an adherence to a gender binary system, whereas the latter are rendered intelligible as “gender outlaws” (p. 238). We see the effect of such “anti-binary models” (Radi, 2019) being manifested in the cissexist assumptions at play in the valuations of these teachers that fail to account for gender expansive understandings in the lives of trans youth who simultaneously straddle and embrace what gets imposed as a simplistic or generalized binary trans identity (Martino et al, 2021). However, it was interesting to reflect on how the normative/subversive framework was employed by teachers in this particular case to expose how gender ambiguity—or, rather, unintelligibility of students who identify as nonbinary/gender fluid—serves as a barrier to addressing their needs because of a lack of teacher knowledge. The problem ultimately is that such a bifurcated binary framing of trans identities sets up a false and oppositional evaluation of two different categories of trans self-identification, which is based on a presumption that occludes a more salient understanding of gender expansiveness that cannot be just confined to those students who identify as nonbinary or gender fluid.

In fact, it was this focus on exposing the effects of the cissexist presumptions at play in perceptions of trans students that precipitated a further nuanced discussion about gender expansiveness in the class. Such discussions were motivated and indeed informed by my reading of Bettcher (2013), for example, who highlights the complexity of trans self-identifications and “how difficult they are to pin down” in that “the very meanings of gender terms are not stable [but] both variable and contested” (p. 389). Johnson (2022) explains that what is required is *expanding* “the definitions and knowledge we hold about what it means to be trans and how trans people navigate our world” (p. 1). Bettcher (2013), for example, explains that in the “beyond the binary model,” trans people are often located “in terms of the categories ‘man’ and ‘woman’ as dominantly understood” (p. 390); this eschews the very transphobic oppression and invalidation of the trans men and women whose self-assigned gender identities are constantly called into question, with demands to provide evidence of, and to account for, their existence (Ahmed, 2016). But as the incident in my class reveals, there is also a cis tendency to pin down and indeed fix the meanings that are invested in certain trans identities that are defined against those students who identify as nonbinary and gender fluid. This issue of trans legibility and recognition strikes at the heart of what my pedagogical intervention has highlighted in opening a space for thinking about resistance and gender expansiveness within the very categories of what it means to be a trans “boy” and a trans “girl,” which cannot be equated with simply adhering to dominant gender binary norms. It also resulted in attention being directed to a sustained interrogation of the effects of cis privilege, which materialized through a set of assumptions and their effects in terms of shaping teachers’ pedagogical understandings of trans students’ identities in schools.

In this respect, Bettcher (2013) argues for an interrogation of “reality enforcement” that “requires recognizing the existence of multiple worlds of sense, worlds in which terms such as ‘woman’ [‘girl’], have different, resistant meanings; worlds in which there exist different, resistant gender practices” (p. 403). In other words, it is important to suspend judgement of trans students who self-identify as boys or girls as simply conforming to dominant gender norms or as necessarily located in any stable or fixed sense within a gender binary system in terms of the meanings they invest in their embodied expression. Rubin (1998), for example, highlights that spaces are needed where trans people can be afforded the opportunity to engage in the productive and creative work of accounting of themselves both “within received categories,” but also in a generative capacity “with the emergence of new discursive categories” (p. 266). For example, there is evidence that many trans youth are creatively and intentionally coupling self-identificatory categories in a variety of seemingly contradictory ways in online community spaces created by and for trans youth; these spaces confound the very imposition of a binary/nonbinary nomenclature. One youth, for example, identifies as both a “transmasculine non-binary person” and mostly “a trans man,” while another identifies as “a nonbinary male” (Martino et al., 2021). As Butler (2001) argues, “recognition” may well operate in ways that seek “to fix and capture us” and, hence, do not necessarily lead to fostering the self-determining capacities of trans students to account for themselves in terms that both defy such binary classifications and are governed by a consideration of “who they are and what their personhood says about the range of human possibility that exists” (p. 30). Connell (2012), for example, argues that one’s trans self-identification needs to be understood within “a larger dynamic of changing gender relations” and that “gender configurations within these structures are multiple, not binary,” which illuminates the reality that “there are always multiple pathways of gender formation as children grow up” (p. 866). This critical insight relates specifically to how Connell (2009) defines the project of gender democratization, which speaks to the epistemic, hermeneutic, ethical, and political concerns that stem from a “beyond the binary” approach to gender justice in schools. Connell conceives of “a strategy of gender democracy” as an alternative to “de-gendering,” which “seeks to equalize gender orders, rather than shrink them to nothing”; she argues that “conceptually, this assumes that gender does not, in itself, imply inequality” (p. 146), and indeed, that identifying as a “man” or a “woman” does not translate necessarily into adhering to a tidy or stable set of dominant gender norms.

## Pedagogical Reflections on Trans Desubjugated Knowledges in the Classroom

In reflecting on the limits of *cissexist pitfalls* and what amounts to a reification of gender binaries in the imposed categorization of trans students has further sensitized me to the urgent need for a trans-informed approach to understanding gender democratization. Such an approach attends to both testimonial and hermeneutic justice in addressing important epistemic matters related to what Stryker (2006) refers to as trans desubjugation. Trans desubjugation entails a commitment to the centering of trans people’s knowledge and interpretation of their own embodied and lived experiences “and of their relationships to the discourses and institutions that act upon and through them” (Stryker, 2006, p. 13). While the need for such an ethical and political commitment is not new, what has struck me and compelled me to write this article in the first place was evidence of traces of an intransigent binary logic seeping into how both educators and researchers think about and/or categorize trans students’ identities (Allen et al., 2020; Bower-Brown et al., 2021). As Mangin (2020) notes, few of the educators in her research “had any specialized knowledge about gender” (p. 11); however, as this incident in my teaching of a 12-week course on gender and sexual diversity highlights, even when teachers are introduced to such knowledge and trans-informed frameworks, they need time, guidance, and space to continue to think through the significance and application of what they are learning via sustained and ongoing critical reflection on their practice. The latter is where trans studies frameworks and a critical trans politics (Spade, 2015) are essential in providing the hermeneutic tools to educate about trans marginalization in institutions such as schools and the academy. Spade (2015), for example, defines a critical trans politics as one that is not so much focused on legal recognition and inclusion of trans people as it is committed to “a distributive analysis” (p. 12)—the ways in which legal and administrative systems of governance “arrange people through categories of indigeneity, race, gender, ability, and national origins to produce populations with different levels of vulnerability to economic exploitation, violence and poverty” (p. 2). In this sense, the critical focus is on system-level governance and “the administration of gender norms” (p. 16) as well as the mobilization of racialized and gender classifications with consequences for further marginalizing trans populations. For example, we see the effects of such governance at the level of individual teachers and researchers through the interpretative and classificatory frames that they employ in making sense of trans students and their identities in bifurcated ways.

Indeed, my own experiences in developing and teaching a trans and queer studies course for educators have further led to an awareness of the threshold knowledges that undergird my own understanding about trans inclusion and gender justice. Such knowledges also have implications for building and scaffolding trans-affirming and professional learning for teachers. Kenway et al. (1997), for example, have recognized the significance of teacher knowledges in gender justice reform in schools:

Knowledge is the core work of schools. Teachers are expected to know their subjects and to know about knowledge, learning and learners. Fundamentally, the teachers’ job is to enable students to become knowledgeable. But what do we mean by “know” and what do we mean by “knowledge”? And what do they have to do with gender reform? What knowledge about gender are our students supposed to acquire and how are they to acquire it? And what do teachers need to know and do in order to assist in this process? What do students do with what we teach them in school? What do we teach them about gender that we don’t know we teach? (p. 66)

Although written more than two decades ago, this statement takes on a particular resonance in light of a trans-infused understanding of the knowledge and critical frameworks that are needed to support teachers in educating about and developing their pedagogical understanding of gender diversity in their classrooms and schools.

## Decolonial Critiques of Gender Binaries and Racialized Cissexism

Such knowledge about gender is informed by both an engagement with trans studies epistemological frameworks and, as I go on to explicate, my own reading of accounts of trans desubjugation. Both serve as hermeneutic resources that inform my understanding of trans phenomena and the lived embodied experiences of trans people, which serves as a basis for scaffolding student learning in my classes about the systemic forces that contribute to trans marginalization. For example, students in the course are required to read chapters from Laing’s (2021) book, which introduce them to the voices and perspectives of Two-Spirit youth and their own self-understandings of their identities. This reading provides important knowledge about Indigenous youth’s “unique relationship to the term”; some feel ambivalent about their understanding of the term, given that it refers to a person who “literally has two spirits” (p. 22). However, Laing demonstrates that her participants had quite complex understandings of Two-Spirit as a term that “is both susceptible to and transcendent of homogenization and misrepresentation” (p. 26)—a phenomenon that she attributes to *cis-heteropatriarchy* as “both a tool and effect of colonialism” (p. 28). In fact, Laing mentioned that one of her participants was critical of hetero-patriarchy, which they claimed has indeed operated in ways that occlude and erase trans people, with heterosexism being used to refer to what are in fact experiences of cissexism that they pointed out was “a huge problem” (p. 54). This “collapsing of queerness and transness” represents a fundamental misunderstanding of differential forms of oppression that are tied to gender and sexuality, which, Laing argues, “is reflective of the ways in which Western systems of classifying gender and sexual diversity within the LGBTQ umbrella disregard the distinctness of gender and sexuality” (p. 33). Indeed, it is a problem that speaks to Radi’s (2019) concerns about an opaqueness that results in a fundamental misrecognition and disqualification of “trans* epistemic subjectivity” (p. 52) (also Bhana, 2022).

In this way, Laing contextualizes her study and the situated knowledges of her participants with respect to their understandings of the term Two-Spirit by drawing attention to the “impacts of colonization on Indigenous modes of gender and sexuality” (p. 18), which have had significant repercussions for Indigenous, queer, trans, and Two-Spirt youth in finding a place of belonging in their communities. However, even though “the forces of colonial racism, homophobia, and sexism cause fragmentation in each participant’s sense of self” (p. 20), Laing shows that the youth were able to move through the effects of these forces over time and to find a sense of belonging in their Indigenous communities. This reading provides an important source of knowledge for students and a space for thinking through what a decolonial approach to addressing gender complexity entails, especially in light of emphatic assertions about a refusal and critique of “the incessant compulsion from non-two-spirit people to explain what two-spirit is” (p. 41) and how the youth were very conscious of not wanting to provide any one definition of the term. Given that the term holds many meanings, Laing highlights that it is important for non-Two-Spirit people to reflect on their *own impulses to fix and objectify* their knowledge of Two-Spirit youth. Rather, what is needed, she argued, is a need to learn from the youth’s own insights about what it means

to be respectful to trans, queer and Two-Spirit youth Indigenous youth . . . a practice which includes but is not limited to: not making assumptions about any one body, gender, assigned sex, or sexuality, and not expecting two-spirit, trans, and queer Indigenous people to explain any details about our lives (or the lives of our communities) to people in order to educate them. (p. 41)

As one of the participants themselves highlighted, having to educate non-Two-Spirit and non-Indigenous people is a draining and time-intensive task that detracts from their own commitment to building community and relationships with other Two-Spirit people.

In light of these comments, some educators raise questions about their role as settler colonial educators in the classroom beyond a mere refusal to make assumptions about Two-Spirit youth and their bodies. This leads to a discussion that Laing herself pivots as vital in interrogating assumptions that apply not only to Two-Spirit youth but to all trans youth. These assumptions require a hermeneutic focus on “cissexism”:

The practice of assuming that everyone (including Two-Spirit people) is cisgender, the practice of assuming that you know someone’s gender based on their appearance, and the belief that man and woman are the only two genders are part of the structure of cissexism that is foundational to the Western worldview. (Laing, 2021, p. 54)

Thus, Laing’s work, with its desubjugated focus on Two-Spirit youth, provides a productive means by which to center a critical focus on how cissexist binary gender systems are central to understanding the forces of settler colonial frameworks that have contributed to enforcing a particular logics of gender and sexuality in schools and in the broader society. As Driskill (2010) notes, “Native-centered and tribally specific understandings of gender and sexuality [are] a way to critique colonialism, queerphobia, racism and misogyny” (p. 69), with Indigenous trans, queer, and Two-Spirit youth in Laing’s study further highlighting the centrality of cissexism as an analytic concept that refuses trans erasure. Such refusal is central to this decolonial critique and to understanding the Imperial project of subjugation of Indigenous peoples and erasure of their cultures: “Indigenous systems of gender and sexuality and the imposition of the cis-heteropatriarchy and gender binary have had deep impacts on Indigenous knowledge systems overall, and have had devastating impacts on the lives of trans, queer, and two-spirit people” (Laing, 2021, p. 109). However, as Laing foregrounds, this critical focus on cissexism enables the sharing of knowledge that fosters a robust understanding of “the interpellation of binary thought into indigenous communities and world views” (p. 29) and how the fragmentation that youth themselves experience is indeed a manifestation of the impact and violence of colonialism. Such a critique, as Driskill (2010) notes, also fosters “a consciousness about the ongoing colonial reality in which all of us living in settler colonial states are entrenched” (p. 71).

Engaging in this reading and then in an extended class discussion with teachers about the forging of such links, which center a critical focus on cissexism, draws attention to the necessity of such spaces for professional learning. Teachers need spaces of ongoing learning that afford them the opportunity to think through their pedagogical practices and what a critical trans politics that is committed to a decolonial critique of gender binary systems means for their practice. Embedded in this directed learning and building of knowledge is the cultivation of teacher professional judgement in the classroom that leads to both a revaluation of one’s assumptions and the sort of curriculum that is needed to foster gender expansive education and racial justice in schools. It leads educators to reflect on the need for further curriculum development and to facilitate learning in their classrooms that attends to enacting a critical trans politics as part of a larger framework of resistance that “refuses empty promises of ‘equal opportunity’ and ‘safely’ underwritten by settler colonialism, racist, sexist [and cissexist], classist, ableist, and xenophobic imprisonment and ever-growing wealth disparity” (Spade, 2015, p. 41). For example, our class discussions in response to Laing’s work raised questions about testimonial injustice. One teacher discussed at length the continuing legacy and impact of the genocide of residential schooling in Canada, with the proliferating discoveries of unmarked graves of children, given that residential school survivors had spoken for years about the existence of these graves and the crimes that led to their creation. In fact, Murray Sinclair, who chaired the Truth and Reconciliation inquiry that released its report in 2015, asserted that the commission had heard from survivors about the children being buried in unmarked burial sites and had called on the government to investigate, but the request was denied (Kirkup & Hagar, 2021; National Centre for Truth and Reconciliation, 2015).

## A Trans-Infused Critique of White Supremacy and Fugitive Justice

This focus on the historical legacy of settler colonialism and the continuance of its reality in our everyday lives created the space to broach broader questions of white supremacy, slavery, and resurgence of anti-LGBTQI+ rhetoric under conditions of far-right extremism. Lavin (2021), for example, comments on how Hirschfield, the Jewish sexologist who opened the first clinic in the world to provide gender-affirming surgery and care for trans people, was targeted by the Nazi state. As a result, thousands of volumes of invaluable material that dealt with same-sex eroticism and gender nonconformity housed in the clinic’s library were confiscated and destroyed. Hirschfield died in exile in Paris in 1935, on the run from the Nazis; however, as Lavin noted, almost a century later, he remains at the heart of attacks by white supremacist neo-Nazi bloggers who target both his LGBTQ activism and work in which he condemned racism. Lavin refers to one particular white supremacist, who referred to how Hirschfield’s “acceptance of ‘degenerate’ forms of sexuality led to his anti-racism,” with the two combined leading to “an intolerable attack on the existence of white people” (para. 10). She also notes how the Drag Queen Story Hour program, a celebration of gender nonconformity since its inception in 2015, has also been a vitriolic target of white supremacist movements, such as the Proud Boys; she witnessed this while observing neo-Nazi chatrooms as part of researching “online white power movements” for her book. Lavin identifies “a manic obsession” with *Drag Queen Story Hour* that materialized in the form of memes, such as one sent to her that featured a drag queen reading to a child accompanied by the following slogan, “And then, one day, for no reason at all, the people voted Hitler into power” (para. 9). In fact, more recently, the Proud Boys stormed a Drag Queen Hour reading event at a library in San Lorenzo, California, hurling slurs at the drag queen; they called her “a groomer” and “a pedophile,” with children being compelled to witness such violence and hate (Bellware, 2022). In fact, it was reported that one of the violators was wearing a T-shirt “with an image of a large AK-47 in the middle, the words ‘Kill Your Local Pedophile’ plastered above it in yellow” (Campoamor, 2022, para. 6). These accounts serve as a powerful source for infusing a critical trans politics into one’s pedagogical repertoire and foreground for educators the ways in which racism, transphobia, and gender phobia are intertwined; and they speak to the need for a coalitional and intersectional approach to gender expansive education. As Spade (2015) argued, it is this overlap in finding solidarity with other struggles—such as those informed by Native Studies and a Two-Spirit critique of colonialism, as well as resistance articulated “through women of color feminism [and] disability justice politics” (p. 12)—that is at the heart of promoting a deeper understanding of trans marginalization and the systemic injustice impacting trans people’s lives.

In this respect, the work of trans scholars from outside the field of education has been vital to me in developing an understanding of the application of trans desubjugation to my own practice and how such knowledge is pivotal in building a teacher threshold knowledge about racial justice and addressing trans visibility/erasure in schools, and the curriculum specifically. Dora Silva Santana (2019), for example, theorizes what she terms *mais viva—*a Brazilian Portuguese term meaning “more alive, alert, savvy”—to provide desubjugated insights into “an embodied knowledge of black and trans resistance” (p. 210). She focuses on one black trans woman, Selen Ravache, who lives in a favela in Rio de Janeiro, as part of her research into learning more about the ways in which black bodies are “imagined, gendered, sexualized, and racialized locally but also connected with a broader experience in the African diaspora” (p. 210). She is interested particularly in how black trans women resist a trans necropolitical social imaginary and existence that is marked by violence—what she defines as “the haunting and material presence of death” (p. 210), and what Snorton and Haritaworn (2013) describe as a form of racialized biopower in which certain factions of the population are marked for death; they are thereby rendered disposable, while others are deemed suitable for “life enhancing investment” (p. 66). Santana is more concerned with how black trans women imagine and live livable lives to care for themselves and their communities to “resist death.” It is this archived sharing of black women’s experiences of what she refers to as “unsubordinated living” that she wants to share with the reader—living that is not defined by a trans necropolitics, but by a “coming together to care for one another” (p. 212) as a trans revolutionary and testimonial act.

Santana met Selen as part of a “trans” support-activist trans group. Selen currently works as a janitor at a hospital and was previously involved in sex work, which enabled her to buy the land she now owns. Her current job, while not well paid, offers benefits and a retirement plan, and, hence, a degree of security. However, we learn of the “everyday adversities” that she experiences in her workplace, which represent a constant *hammering away* at her being as a trans woman (Ahmed, 2016). These involve frequent misgendering and sexual harassment with men trying to trap her into having sex with them at work so they can just beat her to death or get her fired on the grounds that she was harassing them (Santana, 2019). Santana asks her about how she survives under such conditions and avoids being broken at the end of the day. Selen responds with an intensified assertiveness, exclaiming that she “lives life” (Viva a vida!). It is this knowledge about how Selen continues to live life and to heal in the face of such transphobic and racist aggressions that Santana is committed to sharing with the reader. These are the stories she claims are not being told, stories that can generate knowledge and deeper understanding about *fugitive justice*—how black trans women embody fugitivity in “a living of refusal” through occupying “a space between hope and resignation” (p. 215).

Santana draws on Hartman and Best’s (2005) definition of fugitive justice as “a political interval in which all captives find themselves”—it is a space of “mutual imbrication of pragmatic political advance with a long history of failure” (quoted in Santana, 2019, p. 215). Fugitivity also involves “the artful escape of objectification” (Ford, 2015, p. 110, quoted in Santana). Santana (2019) shares how Selen is suing her company for forcing her to use the men’s bathroom at work, and how she has found support in local trans organizations. She shows how Selen continues to live her life in all its everyday mundanity of socializing with friends, going out and enjoying herself, staying connected to trans activist organizations, educating herself about her rights, and maintaining her employment. While Selen simply cannot avoid experiences of cissexist and transphobic aggression, Santana illuminates how she is living life in experiencing a meaningful connection with others and deriving pleasure and joy in living while simultaneously and consciously navigating and anticipating routes of escaping transphobic violence and aggression—what she refers to as “being-alive-savvy” (p. 216). It is about a refusal to “accept a premature death as destiny . . . [and] the imposed gendered and racialized ways of living” (p. 216). It is about an everyday affirmation of one’s being, and agential power that needs to be situated within what Santana refers to as an overall “archive of resistance” (p. 218). In this sense, Santana provides a different way of thinking about black trans women’s experiences that defy the ways in which both transness and blackness are defined and understood.

Thus, Santana deploys Selen’s narrative account of her own embodied existence of living her life to both interrupt and interrogate the various ways in which “visibility, legibility and intelligibility structure a grid of imposed value on the lives and death of black and brown trans women” (Snorton & Haritaworn, 2013, p. 68). Selen’s narrative represents what Snorton (2017) refers to as “the return of the gaze that negotiates at every point a space for living” that allows for the willful naming of “a counter-power and counter-mythology” (p. 143), which “become[s] ways to read race and gender as indexes of power’s circulation and as instantiations of the ways discourse recursively presses flesh into bodies of meaning over time” (p. 144). As Santana argues,

for Selen as for so many of us, refusing to lose her patience, her sanity, her professionalism, her job, her joy is an embodied praxis of fugitivity . . . where being *mais viva* constitutes a situated experience, that contingent movement that is trans and those routes of escape that also define blackness. (p. 218)

Snorton (2017) provides additional examples of such “archives of resistance” that enrich a pedagogical repertoire for building a threshold knowledge about black trans life. Indeed, the archival accounts of trans lives lived that he traces in his work exceed the traps of trans representation and visibility that are defined by a liberal embrace of diversity and inclusion (Martino, Kassen, & Omercajic, 2022). Gossett (2017), for example, argues that “one of the traps of trans visibility is that it is premised on invisibility: to bring a select few into view” (p. 183), with its implications for reinforcing oppression in further perpetuating trans erasure in its failure to account for the “psychic life of racism in the afterlife of slavery” (p. 184). They assert that the “violence of colonialism and racial slavery, through which black, queer, and/or trans identifies have been forged cannot be addressed through the politics of trans visibility” (p. 183). Snorton’s genealogical accounts of blackness and transness within the context of racial slavery are a powerful testament of this. He provides archival evidence of how Sims, who became known as “the father of modern gynecology” (p. 20), conduced experimental interventions on black women’s bodies; these interventions rendered them objects “in the production of medical knowledge that critically disavowed chattel slavery as a constitutive grammar to express sex and gender as effects of racial science” (p. 41) and resulted in such unbearable suffering for these women. He provides details of how in 1930s, only black cadavers were used to teach about anatomy, further highlighting how black bodies were turned into “fungible flesh.” This was also illustrated in publications such as *Great Moments in Medicine*, published in 1960, in which “slave girls” were represented as being subjected to the medico-clinical gaze of Sims and his fellow physicians within the economy of what Snorton referred to as “plantation medicine” (p. 50).

Snorton’s (2017) argument is that such archival accounts of racial signification, and indeed the objectification and fungibility of “captive flesh” within the context of slavery, serve as “a critical genealogy for modern transness as chattel persons gave rise to un understanding of gender as mutable and as amenable form of living” (p. 57). He provides further accounts of such fungibility in his book; for example, he details what he refers to as fugitive narratives in which cross-dressing practices in the form of *passing* as the opposite sex served as a means by which fugitives disguised as men or women were able to plan escape routes North out of the captive violence of chattel slavery in plantations in the South. Snorton discusses various iterations of the fugitive journey of Ellen Craft, who was read as a white woman and dressed in a man’s clothing so that she could masquerade as “the master,” with her husband presenting as her servant. It is in this capacity that Snorton elucidates not only “how blackness as a condition of possibility made transness conceivable in the twilight of slavery,” but also how the persistence of a negation of black life and transness in the aftermath of slavery was a central component of modernity marked by experiences of “unfreedom” (p. 142) and “the violence of erasure” (p. 144).

However, Snorton (2017) argues that such erasure needs to be viewed not so much as an absence, but as “an animating presence,” given the trans black lives that were lived in the shadows of the racial order that included Christine Jorgensen, a white trans woman, “as an exceptional figuration of trans embodiment” (p. 139). Indeed, Jorgensen achieved a significant degree of notoriety and celebrity status in the 1950s after undergoing gender-affirming surgery. Snorton provides critical insights into how such spectacularized representations of Jorgensen as the embodiment of “transsexual freedom” in the mainstream press at the time were “rendered visible through her whiteness” (p. 160) within the context of Cold War ideology in the United States and increasing attention to decolonial resistance movements in other nations. He makes sense of Jorgensen’s rise to notoriety under these conditions of “colonial imperial authority” in the United States, which permitted a particular instrumentalization of a white narrative of personal triumph and individual freedom that was born out of “a constitutive disavowal of the nation’s foundational logic of white supremacy” (p. 161).

Snorton (2017) illustrates this phenomenon by providing narrative accounts of “racialized unfreedom” of black trans people’s lives, both prior to and after the emergence of Jorgensen as a celebrity figure. For example, he documents the accounts of two black trans women (and others)—Carlett Brown and Ava Betty Brown—in the aftermath of Jorgensen’s postoperative notoriety. They were subjected to police harassment and did not have the same sort of access as Jorgensen. In seeking gender-affirming surgery in Denmark, Carlett was told that she would have to renounce her U.S. citizenship (which she did) in order to receive treatment from the same specialist who supervised Jorgensen’s surgery, which was not permitted in the United States. However, Carlett’s plans were thwarted because she was imprisoned for wearing women’s clothing in public, and she was not able to raise the money for her bail. Under such structural conditions of police harassment, Carlett asserted the “right to life liberty and the pursuit of happiness” for *female impersonators*, “especially when they are minding their own business” (p. 161). Snorton highlights how Carlett’s experiences of police harassment and conditions of existence illuminate a racialized form of unfreedom that ruptures the ways in which Jorgensen’s story was instrumentalized as an instantiation of progress and individual freedom in the United States—one that is indeed “shored up by whiteness” and a constitutive disavowal of the nation’s foundational logic of white supremacy” (p. 161). Furthermore, Ava Brown, who was also arrested for wearing women’s clothes, asserted in court, upon being asked to account for herself as a woman, that “all of her friends and business acquaintances knew her as Ava Brown” (p. 162); everything she owned was in this name, leading her to declare, “If I am a man. I don’t know it” (p. 162). In this agential capacity, Snorton argues that, like Carlett, Ava was able to declare herself a black trans woman, which called into the question the reality that gender transformation can only be attained as a means of legal and medical intervention and the recognition that it supposedly ensures. Hence, Snorton argues that Ava Brown’s assertion serves to provide insight into “black sociality” as a “site for gender articulation” that speaks to “knowledge systems unrecognized by colonial authority” (p.162).

It is in this sense that Gossett (2017) argues that “blackness ruptures trans representability, respectability, and visibility” in drawing attention to “the grammar of “cisgender” [as] lack[ing] the explanatory power to account for the colonial and anti-Black foundational violence of slavery and settler colonialism through which the gender and sex binary were forcibly rendered” (p. 185), which is exemplified by both Laing (2021) and Snorton (2017). Gossett also highlights the role that black feminism has always played in the challenges that black feminists have mounted to “categories of binary gender and of medically assigned binary sex, while continuing to call for liberatory forms of abolition” (p. 186). This also speaks to the critical trans political lens that Santana (2019) brings to her account of Selen’s life. The work of these scholars has been a powerful pedagogical resource in my teaching of this graduate course, particularly with respect to centering the lived experiences of black trans women and in excavating trans narratives from historical archival sources that illuminate “the colonial racialization of blackness” (Gossett, 2017, p. 186); these sources are pivotal in building teacher threshold knowledges about racial justice and trans visibility. Contextualized and situated knowledges (Haraway, 1988) derived from these hermeneutic and testimonial sources exceed the limits of facile commitments to trans inclusion, recognition, and representation. In fact, learning from these sources gestures toward a vital need for curriculum development committed to creating spaces for learning about trans lives that are contextualized within a broader examination of the interlocking systems of power and privilege in which all of our lives are enmeshed and intertwined (Beauchamp & D’Harlingue, 2012). This is particularly salient when thinking about questions pertaining to colonial authority with respect to centering a critical focus on the racialized and cissexist legitimation of gender binary systems and their continued impact.

By incorporating Selen’s narrative into a pedagogical repertoire within the context of teaching my course, for example, a further space for addressing questions related to both the significance of black trans feminist frameworks and trans desubjugation as a source of knowledge for fostering an understanding of fugitive justice is created. This sort of sharing leads to a consideration of Snorton’s work on the racial history of trans identity and experience and serves as a directed application to thinking about the pedagogical implications of educating about black trans life within the context of slavery and white supremacist ideologies. Drawing on the hermeneutic sources provided by Laing (2021), Santana (2019), and Snorton (2017) in my own teaching and sharing the preceding narratives with my students have enabled me to think through and to help teachers think through what a trans studies approach and critical trans politics might mean in more concrete terms with respect to reflecting on the curriculum as a space for enacting gender-expansive education. This work from outside the field of education raises crucial questions about the vital importance of engaging with trans studies scholars and to accessing this desubjugated knowledge in the classroom in working with educators to support their learning and developing understandings of what it means to enact a critical trans politics in their classrooms and schools—one that is committed to testimonial and hermeneutic justice.

As a means of scaffolding this work, I share resources such as the recently published picture story book *Sylvia and Marsha Start a Revolution* (Ellison, 2021). The book introduces children to Marsha P. Johnson and Sylvia Rivera, two revolutionary trans women of color who were at the forefront of, and one of the driving forces behind, the Stonewall rebellion—revolutionaries of whom many of my students seemed to have no knowledge. I also introduce them to Stryker’s (2005) *Screaming Queens* documentary, which focuses on the experiences of trans women and drag queens who stood up to and fought police harassment at Crompton’s Cafeteria in San Francisco’s Tenderloin district three years before the irruption of the Stonewall riots. Such resources provide insight into agential capacities of trans people that defy a necropolitical emphasis on reducing black trans lives to representations of death and disposability, while simultaneously exposing the racial, economic, and cissexist systems that structure the terms by which trans people are subjugated and denied access to life-enhancing resources. Starting with the required reading by Laing (2021) as a means by which to create a space for discussing racialized cissexism and the settler colonial logics that are at the heart of gender binary systems serves as a foundational basis for extending discussions about colonial authority and domination into the realm of black trans lives lived under conditions of slavery and continuing white supremacy. It also serves as a foundational basis for discussing the contextualization of the anti-trans rhetoric and white supremacist ideologies that continue to unravel in disturbing ways at this particular moment in history, marked by a resurgence of far right extremism (Lavin, 2021).

## Implications

In stepping back from my own reflections on a trans studies–informed pedagogical approach in this article, what is striking is how accounts of the students in my class and what they learned receded into the background as I moved beyond the critical incident. This is a particular limitation of what is presented here and has significant implications for the work that is needed moving forward, particularly with respect to supporting teacher professional learning and the lessons to be learned from the sense they are making as they think through the implications of the course for their practice. In other words, what is needed is a redirected focus away from myself to a centering on the learning and unlearning that the students are doing as we struggle together in the pedagogical space of the course and that is encapsulated by the critical incident out of which this article grew. This need to learn more from the students themselves with respect to their engagement in the course is illuminated by what transpired after reading their final assignments. The assignment required students to reflect on their practice, given their understanding of trans studies frameworks. It was quite open-ended in this sense, but students were encouraged to reflect in a more focused manner on classroom practice/life in schools and to consider what the course meant for addressing gender justice, particularly with respect to fostering student learning about gender expansiveness. For example, one history teacher wrote about how the course had helped her to develop and extend a current unit of work on LGBT history in her Grade 11 class—inserting both *Screaming Queens* and a focus on the activism of Marsha P. Johnson and Sylvia Rivera—in light of attempting to take up questions of both gender and racial justice in raising questions about trans visibility and erasure with her students. This teacher also reflected on incorporating and extending a focus on Hirschfield within the context of teaching about World War 2. However, as just one example, the elementary school teachers chose not to take up a critical reflection on *Sylvia and Marsha Start a Revolution* despite my encouragement to do so, and despite the attention to gender, sexuality, and racial justice throughout the course. The myriad possible reasons for this are beyond the scope of this article (e.g., the construction of assignment itself, the need for more sustained and scaffolded engagement with picture storybook resources that target elementary teachers specifically, fear and/or lack of confidence or self-efficacy, given their own particular context and/or the broader context of anti-trans rhetoric and resurgent far-right extremism). Regardless, this absence does highlight the need for centering the voices and experiences of my own students and to pivot them as powerful sources of knowledge for learning more about what trans studies frameworks mean for our practice.

The article also raises questions about the sustainability about the ongoing provision of PD, especially given that what is needed are opportunities for educators to work through what trans studies frameworks and racial justice mean for their practice. The salience of this necessity is heightened under conditions of constraint and lack of PD provision in schools and teacher education, which lead to responsibility for this sort of learning falling on the shoulders of individual educators themselves (Martino, Omercajic, & Kassen, 2022). For example, the educators who enrolled in our program did so out of their own commitment to educating themselves, and they were passionate about wanting to create more equitable conditions for students in their schools, especially for those from minoritized communities. This issue of self-reponsibilization further illuminates the structural conditions of hermeneutic marginalization, which are at the heart of this problem of fostering trans intelligibility in schools, and which Fricker (2007) referred to as “the whole engine of collective social meaning [which is] effectively geared to keeping . . . experiences [obscured by cissexism and racial ideologies of white privilege] out of sight” (p. 153). Given these structural constraints, what is evident is that support for educators is even more necessary in the current context of resurgent anti-LGBT rhetoric and legislation under conditions of far-right extremism fueled by white supremacist ideologies. What I have learned from educators themselves is that this sort of professional learning is instrumental in enhancing their overall self-efficacy and indeed savviness in navigating the system and conditions of constraint with respect to dealing with backlash and pushback in their own communities.

## Conclusion

This article emerged out of ongoing concerns about the need for a trans studies approach to gender-expansive education and trans inclusion in schools. Questions related to epistemic justice and trans desubjugation were identified as a foundational basis for thinking about the application of a trans studies approach and what a critical trans politics entails with respect to a consideration of building teacher threshold knowledges that are central to addressing gender and racial justice in schools. Nicolazzo (2017), for example, noted that despite “the increase in trans* visibility . . . trans* women, trans* people of color, and trans* - and trans* people with disabilities—and trans people with the aforementioned marginalized identities—continue to occupy incredibly precarious positions across social contexts” (p. 212). In this respect, this article represents a mapping of my own attempt to think through the terms of a trans pedagogical commitment to gender and racial justice that goes beyond a mere politics of visibility and representation in creating a space of sustained learning for educators that fosters a critical reflexivity about cissexism, trans erasure, and racial justice. However, my own experience of supporting the professional learning of educators highlights that creation of such spaces needs to be sustained over time. In short, scaffolded professional learning opportunities are needed for educators to build their knowledge and pedagogical application of a trans studies approach to addressing gender and racial justice in their classrooms and schools, particularly with respect to the cultivation of teacher judgment.

What is clear, as the critical incident illuminated, is that in the absence of embracing a trans-informed approach to thinking about gender diversity, with its critical interrogation of racialized cissexism, there is a risk of educators relying on the deployment of a bifurcated categorization of trans students’ identities. In this respect, and as I have exemplified throughout this article, the contribution of trans studies scholars has been an indispensable resource for me in this endeavor. The frameworks that trans studies affords and the knowledge generated by Two-Spirit scholars such as Laing (2021), and black trans scholars such as Gossett (2017), Santana (2019), and Snorton (2017), have a particular pertinence to and salience for educators and researchers in thinking through the political and ethical terms of their practice, particularly with respect to the provision of the necessary hermeneutic resources and tools for addressing gender and racial justice. Central to this project is a need for an interrogation of gender hierarchies and cissexism, as well as a decolonial critique of bifurcated gender binary systems of thought. Such critical frameworks are central to a pedagogical and research commitment to envisaging a trans imaginary (Martino, 2016) that exceeds both cissexist pitfalls and the terms of living life that is defined and delimited by necropolitical violence.

## Notes

1. While our work has focused on the application of trans studies in schools, it is important to acknowledge the contribution of trans scholars such as Nicolazzo (2017, 2021) and Simmons (2017) specifically with respect to the application of trans studies in postsecondary education.

2. My understanding of critical incidents stems from my reading of Tripp’s (1993) approach to fostering professional learning of teachers. A critical incident can emerge out of reflection on one’s routine teaching through sustained documentation of one’s observations in a professional learning journal, or it can be an unexpected event, action, or response from a student in class that warrants further analysis. Identifying and reflecting on critical incidents are central to generating deeper understandings of one’s pedagogical practices and their effects in enhancing and fostering student learning.

3. The course is part of a professional program devoted to addressing equity and social justice education. It is a two-year cohort-based program. My course is offered in the second year, after students have already taken introductory courses in anti-oppressive education, Indigenous education, and anti-poverty education, and where there is an integrated focus on intersectional approaches to addressing equity. The rationale for the structure of my course is related to addressing the problem of conflating “queer” with “trans,” which, as trans scholars have highlighted, has resulted in a failure to understand and to do justice to trans people’s own embodied self-understandings of their personhood (Radi, 2019; Raun, 2014). Students, therefore, are introduced to a range of analytic concepts, such as heteronormality, heterosexism, homophobia/cisnormativity, cissexism, and transphobia, and provided with opportunities to apply them to their developing understandings of gender and sexual diversity in schools.
